# Establishing the global working group on Reproductive and Developmental Environmental Health (RDEH): practicum of a global resource

**DOI:** 10.1097/GRH.0000000000000018

**Published:** 2018-08-16

**Authors:** Allison M. Power, Tracey J. Woodruff, Jeanne A. Conry, Linda C. Giudice

**Affiliations:** a Department of Obstetrics, Gynecology and Reproductive Sciences, University of California, San Francisco, San Francisco, CA; b Program on Reproductive Health and the Environment, UC San Francisco, San Francisco, CA; c Environmental Health Leadership Foundation, Granite Bay, CA

**Keywords:** Reproductive health, Developmental health, Environmental health, Environmental exposure, International Federation of Gynecology and Obstetrics, FIGO, Reproductive and Developmental Environmental Health work group, RDEH

## Abstract

Advocacy, training, and research for the prevention of adverse health effects from environmental exposure are the major foci of the International Federation of Gynecology and Obstetrics (FIGO) Reproductive and Developmental Environmental Health (RDEH) Working Group. A critical need for a consistent global effort to address these threats to human reproductive and developmental health was identified after the publication of influential committee opinions (*ACOG, Obstet Gynecol*, 2013; FIGO, *Int J Gynaecol Obstet*, 2015) and the assembling of the FIGO 2015 Pre-Congress Workshop, “Summit on Shaping Our Planetary Legacy: Setting an Agenda for Environmental Reproductive Health.” RDEH’s goals are to convene experts of diverse backgrounds to generate evidence-based knowledge and propose solutions to the increasing threats of harmful environmental chemicals to global human reproduction and development by prevention of harm through education, research, and advocacy. This practicum is a guide for establishing a global task force, such as FIGO RDEH, for developing collaboration among experts across disciplines and time zones, and for building accessible and vital databases.

Over the past 15 years, an expanding body of evidence has demonstrated the role of harmful environmental exposures on human health^[1–3]^. Whether scientists are reviewing increased rates of cancer, neurodevelopmental disorders, pregnancy complications, or birth defects, evidence-based research shows the significance of harmful environmental exposures on these outcomes globally. Exposures that occur during sensitive periods of reproduction and development are of particular concern. In 2013, ACOG published its Committee Opinion, *Exposure to Toxic Environmental Agents*, which was developed in response to science and policy concerns about these exposures to reproductive health^[1]^. In further recognition of the need for a consistent global effort to address threats to human reproductive and developmental health, the International Federation of Gynecology and Obstetrics (FIGO) put forward its opinion, *Reproductive Health Impacts of Exposure to Toxic Environmental Chemicals*^[3]^. The FIGO Opinion concludes with four recommendations to reduce and prevent exposure to toxic chemicals globally. The 4 recommendations are: (1) advocate for policies to prevent exposure to toxic environmental chemicals; (2) work to ensure a healthy food system for all; (3) make environmental health part of health care; (4) champion environmental justice. FIGO is well-situated to remark on the role of harmful environmental exposures on reproductive and developmental health as a professional organization that unites obstetrical and gynecological associations from over 130 countries and whose mission is to improve the health and wellbeing of women and children worldwide.

Momentum for the FIGO Opinion paper was accelerated at the FIGO Pre-Congress Workshop at the triennial meeting in Vancouver, October 2015, “Summit on Shaping Our Planetary Legacy: Setting an Agenda for Environmental Reproductive Health.” This interactive workshop brought together leaders of reproductive health professional societies from around the globe to develop a plan of action to address exposure to harmful environmental chemicals. Together, workshop participants developed a preliminary action plan for research and policy. A critical need for a FIGO Working Group to carry this agenda forward and develop new directions was identified. FIGO soon established the Reproductive and Developmental Environmental Health (RDEH) Working Group, whose charge was to identify and engage stakeholders, as well as set the framework to tackle this complex issue. RDEH’s goals are to convene experts of diverse backgrounds to generate evidence-based knowledge and propose solutions to the increasing threats of harmful environmental chemicals to global human reproduction and development by prevention of harm through education, research, and advocacy. Long-term success will be measured by global awareness, as we have seen with climate change. Implementation of best practices and continued research on the effect of minimizing harmful exposures on reproductive and developmental outcomes are important goals for RDEH and its stakeholders for the future.

Soon after FIGO Leadership established the RDEH Working Group, subcommittee members—chosen by Co-Chairs Giudice and Conry—received and accepted formal invitations to join the Working Group. Following these invitations, the RDEH Secretariat, Ms Allison Power, created a leadership chart to ensure the subcommittees (Advocacy, Training and Capacity Building, and Research) remained diverse in gender and geography. Ms Power then worked with Drs Giudice, Conry, and Woodruff (the “Operations Team”) to identify the frequency of meetings and the best format for collaborating with members worldwide. The WebEx platform hosted by the University of California, San Francisco (UCSF), with the ability to call in through a computer’s internet access, was deemed the most user-friendly and cost-efficient tool. In an effort to work with various schedules and time zones, monthly polls with date and time options were sent globally. All members received calendar invitations, e-mail reminders, and a link to relevant time zones using an online time and date world clock converter. The FIGO RDEH Working Group continued to develop through the Secretariat’s role in coordinating these teleconferences, creating and updating online assets, troubleshooting IT issues, and providing support for social media campaigns, funding applications, and collaboration requests.

For the first 6 months, FIGO RDEH held 4 web-based meetings per month, 1 per subcommittee and 1 combined meeting for all members. The Secretariat opened the calls on WebEx 15 minutes before the meeting in order to assist members. After connectivity problems beyond the group’s control, the Operations Team switched to ReadyTalk, an audio conferencing service. Toll-free international numbers, as well as operator call-in options for those unable to dial-in were provided. The Secretariat kept teleconference attendance records, while also taking detailed minutes with highlighted action items. Post-meeting follow-up to the whole group included edited minutes by Drs Giudice and Conry, as well as the Subcommittee Leads, and links to any new additions on the FIGO RDEH Box asset folder (https://ucsf.box.com/v/FIGORDEH).

Ms Power created—and continues to maintain—the FIGO RDEH asset folder on Box; this includes 4 main subfolders: Operations, Resource Depository, Advocacy and Outreach, and Research Inventory. Each subfolder is accessible to all members to provide a way for them to access complex environmental content for their own lectures, conversations, social media posts, etc., as a part of the FIGO RDEH dissemination goal. The Operations folder is an archive of minutes, agendas, meeting recordings, the leadership chart, the overall goals of FIGO RDEH as well as its subcommittees main foci, budget drafts, funding research, proposals, grant applications, letterhead templates, and other administrative documents. The Resource Depository includes slide decks, articles, short films, declarations and meeting summaries, an inventory of presentations available for use (Appendix A2), policy statements, teaching guidelines, and other tools to aid in the creation of more RDEH-teaching material. The Advocacy and Outreach folder includes a FIGO RDEH lecture repository, upcoming conferences with abstract deadlines, social media campaign information and template posts and graphics, potential speakers and collaborators for the “Train the Trainers” initiative, and more. The Research Inventory folder includes the list of named RDEH research centers worldwide and their annual reports when available.

In addition to managing and updating these subfolders and fielding recommendations for the various inventories on Box, the Secretariat supports special campaigns put forth by FIGO RDEH and other organizations. For example, during the 2017 Earth Day Social Media Campaign, the Secretariat reached out to over 30 organizations with ready-made tweets, the @FIGOHQ Twitter handle, and concise instructions on how to support the Earth Day initiative. In follow-up of the Earth Day campaign, through e-mails sent by Ms Power on behalf of Drs Giudice and Conry, over 25 key FIGO Leaders received a letter of collaboration asking for invitations to annual meetings to present on RDEH topics and push forward the “Train the Trainers” initiative. The Secretariat also plays a key role in drafting grant and foundation applications, tracking deliverables, and ensuring timely submissions of internal FIGO reports. The ultimate role of the Secretariat is to hold the Working Group members accountable and help them focus on the FIGO RDEH mission to spread awareness, promote education and research, and inform policy on reproductive and developmental health impacts of exposure to harmful environmental chemicals.

Since its inception, the FIGO RDEH Working Group has identified a global membership of dedicated scientists and physicians who have worked remotely to further FIGO’s vision (Appendix A1). Over its initial 3 years, 27 RDEH members have volunteered their time working collaboratively and meeting regularly by teleconference to develop plans for advocacy, training and capacity building, and research that address harmful environmental exposures on reproductive and developmental health.

RDEH has identified 3 subcommittees of experts to spread awareness, promote education and research, and inform policy on the reproductive health impacts of exposure to harmful environmental chemicals:

- Advocacy—the Advocacy Subcommittee focuses on promoting the need to address the links between maternal and child health (MCH), noncommunicable diseases (NCDs), and environmental exposures in a cohesive manner and determine innovations within health systems and public policy. It works with other advocacy organizations and stakeholders such as the World Health Organization (WHO), United Nations Environment Programme (UNEP), and International POPs (persistent organic pollutants) Elimination Network (IPEN). The Advocacy Subcommittee also focuses on strategies for public policy changes and advocacy, as most exposures to environmental chemicals require innovative and enforceable public policy directives. In addition, outdoor and indoor pollution comprise a global issue that requires a strong, evidence-based global response.
- Training and Capacity Building—the Training and Capacity Building Subcommittee works with the FIGO Committee on Capacity Building in Education and Training and members of national medical and other professional associations to develop curricula and training materials to implement the RDEH-initiative for all levels of health care providers, stakeholders, and advocacy groups. The group supports twinning, wherein one national association with greater capacity within or outside the region can help another national association to increase capacity. Educational courses and lectures to implement the RDEH-initiative have taken place at regional and national conferences. FIGO RDEH also keeps a resource depository of slides and curriculum, as well as social media strategy information and posts to share around the globe as part of the Training and Capacity Building Subcommittee goals.
- Research—the Research Subcommittee is working with national associations, academic institutions, and other FIGO Committees to promote, encourage, and support collaborative research in the field of implementation and operational science and clinical and basic science. Research priorities identified in the RDEH-initiative as well as other relevant areas receive due attention.

## Challenges

Identifying partners with common purpose was initially a challenge for the RDEH Working Group, which benefitted from extensive networking and perseverance, and while these relationships have been established, it continues to be a work in progress. Furthermore, establishing evidence-based information about harm to human health by environmental contaminants is a time-consuming process that requires extensive resources in personnel and finances. Numerous groups are advancing these efforts through funded grants from federal and local institutions and foundations, as regulation of these pollutants and information about health effects are limited in most countries around the world. Altering physician practices and informing public policy requires years to effect change, and thus a long-term perspective is essential, along with aiming for and appreciating “small gains” along the way. Furthermore, providing minsters of health with evidence-based, preventive strategies requires intensive culling of evidence-based information, systematic synthesis of standards to guide policy and practice, and advocacy. Getting the word out about environmental pollutants and preventive strategies to health professionals, those in training and to the lay public, adopting these in real time, and measuring outcomes are major endeavors. Sustainability of RDEH, a global resource, is one of our goals and challenges for the future. Thus, our effort to be an integral part of educational programs for health care providers and other professionals around the globe is paramount, as there is much at stake for the health and wellbeing of this and future generations.

## Appendix

### A1. Conferences and significant events

- 2005: In general, patients, scientists, policy makers, funders, and healthcare providers rarely have an opportunity strategize together about impacts on all stakeholders. To overcome this in the emerging field of reproductive environmental health, the Vallombrosia Workshop was convened in Menlo Park, California. The workshop, “Understanding Environmental Contaminants and Human Fertility: Science and Strategy,” was organized by Dr Linda Giudice then at Stanford University and Ms Alison Carlson of the Collaborative on Health and the Environment (CHE).
- 2007: The content and format of the Vallombrosia Workshop were subsequently expanded to a multidisciplinary summit, the “UCSF-CHE Summit on Environmental Challenges to Reproductive Health and Fertility,” in San Francisco, California. This resulted in publication of a scientific review^[4]^ and a lay monograph, “Challenged Conceptions: Environmental Chemicals and Fertility.”
- 2007: Dr Giudice founded the Program for Reproductive Health and the Environment (PRHE) at the University of California, San Francisco (UCSF), Department of Obstetrics, Gynecology and Reproductive Sciences, with funding through the Dean of the UCSF School of Medicine. Dr Tracey Woodruff was recruited as Director of this new program and continues to this day.
- 2008: A report on the Summit, “Shaping Our Legacy” and the scientific proceedings that covered the state of the science in male and female reproductive tract and fertility compromise with a focus on humans was published as an online Supplement in Fertility and Sterility in February 2008^[4]^, and are available, respectively, at: https://prhe.ucsf.edu/sites/prhe.ucsf.edu/files/shapingourlegacy.pdf and http://www.fertstert.org/issue/S0015-0282(08)X0190-6.
- 2013: Evidence-based Joint Committee Opinion from ACOG (American Congress of Obstetricians and Gynecologists) and ASRM (American Society for Reproductive Medicine) on how harmful environmental chemicals in the environment harm our ability to reproduce, negatively affect pregnancies, and affect long-term health. This sentinel publication was released in 2013 and has provided guidance for state and federal positions on legislation, advocacy, and education in the United States^[1]^.
- 2014: Proposal and scientific opinion were submitted to the International Federation of Gynecology and Obstetrics and published in 2015^[3]^.
- 2015: The XXl World Congress of FIGO, with a pre-Congress of invited physicians to identify global issues of environment and health. Dr Giudice gave the Markku Seppala Ovidon lecture, Keynote address, and the pre-Congress generated global interest in women’s health and exposure to harmful environmental chemicals.
- 2016: Members of the RDEH Working Group participated in Project TENDR: Targeting Environmental Neurodevelopmental Risks. Project TENDR has brought together international scientific, clinical, and public health leaders to address the impact of multiple environmental exposures on neurodevelopment and the long-lasting effect on children’s health. This group has identified examples of pollutants and exposures that increase the risk of serious neurodevelopmental effects.
- 2016: Global Conference on Maternal Infant Health: Strategies and critical issues for ameliorating global health of mother, fetus, and newborn. RDEH Working Group leadership presented impacts regarding environmental exposure and climate change.
- 2016: Members of the Working Group met with the WHO to formulate a plan regarding shared interests in the health of children, based on the risks of peri-conception and prenatal environmental exposures.
- 2017: RDEH Working Group plan was presented to the Executive Board of FIGO and received enthusiastically, with the request that they meet in person to present a proposal in 2018 to the World Congress.

### A2. Presentations resource depository

1. Science Communication: Communicating the Value of Basic Science Research.
2. Environmental Toxicants and Endocrine Disruptors: Effects on Reproductive Outcomes.
3. International Federation of Gynecology and Obstetrics RDEH Orientation.
4. FIGO Reproductive Health and the Environment.
5. FIGO SLIDE TEMPLATE RDEH.
6. Global Reproductive Health and the Environment: What does the evidence say?
7. How Safe is the Environment? Contaminants & their Effect on Reproductive Health.
8. Physicians and Climate Solutions.
9. RDEH: Ad Hoc Committee on Health and the Environment.
10. Reproductive Environmental Health: From Policy to Patients.
11. The Society for Maternal-Fetal Medicine (SMFM) Scientific Forum on Substance Abuse & Toxicology.
12. Update on the Hidden Reproductive Health Hazards of Environmental Toxins.
13. Environment, Hazards and Climate Change.
14. La Federación Latinoamericana de Sociedades de Obstetricia y Ginecología (FLASOG): Impact of the Environment on Reproductive and Developmental Health.
15. La Federación Latinoamericana de Sociedades de Obstetricia y Ginecología (FLASOG): El Impacto del Medio Ambiente en la Salud Reproductiva.
16. Sri Lanka College of Obstetricians & Gynaecologists (SLCOG) Golden Jubilee Congress 2017: Women’s Preventive Services Initiative (WPSI).
17. All India Congress of Obstetrics & Gynaecology (AICOG) RDEH: Sixty-first All India Conference on Obstetrics and Gynaecology.
18. Early Avoidable Environmental Exposures (Parts 1 & 2).
19. Japan Society of Obstetrics and Gynecology (JSOG) Environmental Toxicants Affecting Human Reproductive Health: What is the Evidence?
20. Japan Society of Obstetrics and Gynecology (JSOG) Precision Medicine: New Frontiers in Women’s Health.
21. RDEH: For Capacity Building and Education Training.

## References

[1] American College of Obstetricians and Gynecologists Committee Opinion No. 575. Exposure to toxic environmental agents. Obstet Gynecol 2013;122:931–5. 24084567 10.1097/01.AOG.0000435416.21944.54

[2] Landrigan PJ, Fuller R, Acosta NJR, The Lancet Commission on pollution and health. Lancet 2018;391:462–512. 29056410 10.1016/S0140-6736(17)32345-0

[3] Di Renzo GC, Conry JA, Blake J, International Federation of Gynecology and Obstetrics opinion on reproductive health impacts of exposure to toxic environmental chemicals. Int J Gynaecol Obstet 2015;131:219–25. 26433469 10.1016/j.ijgo.2015.09.002 PMC6663094

[4] Woodruff TJ, Carlson A, Schwartz JM, Proceedings of the Summit on Environmental Challenges to Reproductive Health and Fertility: executive summary. Fertil Steril 2008;89:e1–20. 18308046 10.1016/j.fertnstert.2008.01.065

